# In Vitro Antimicrobial Activity and In Vivo Intestinal Barrier Regulation by *Commiphora leptophloeos* Bark Extract in 5‐Fluorouracil–Induced Mucositis

**DOI:** 10.1002/cbdv.202503251

**Published:** 2026-04-01

**Authors:** Stphannie Jamyla de Araújo Barbosa, Renato Dantas‐Medeiros, Susana Barbosa Ribeiro, Amanda Silveira da Silva, Lucas Vinicius Souza de Carvalho, Rafaela Torres Dantas da Silva, Gerlane Coelho Bernardo Guerra, Raimundo Fernandes de Araújo Júnior, Nizyara Costa da Silva, Raphael Victor Silva Andrade, Gabrielle Marques de Paiva, Silvana Maria Zucolotto, Rafaela Alcindo Silva de Sousa Fé, Maria Laura de Souza Lima, Raphaël Grougnet, Sergio Ortiz, Ana Caroline Zanatta, Aurigena Antunes de Araújo

**Affiliations:** ^1^ Postgraduate Program in Pharmaceutical Science Federal University of Rio Grande Norte Natal Brazil; ^2^ Department of Pharmaceutical Sciences Federal University of Rio Grande Norte Natal Brazil; ^3^ Department of Biomedicine Federal University of Rio Grande Norte Natal Brazil; ^4^ Postgraduate Program in Pharmaceutical Science Department biophysical and Pharmacology Federal University of Rio Grande Norte Natal Brazil; ^5^ Postgraduate Program in Health Sciences, Postgraduate Program in Functional and Structural Biology UFRN, Department of Morphology Federal University of Rio Grande Norte Natal Brazil; ^6^ Postgraduate Program in Oral Science Department of Dentistry Federal University of Rio Grande Norte Natal Brazil; ^7^ Postgraduate Program in Pharmaceutical Science Postgraduate Program in PPgDITM Department of Pharmaceutical Sciences Federal University of Rio Grande Norte Natal Brazil; ^8^ Faculté de Pharmacie Université Paris Cité Paris France; ^9^ UMR 7200 Therapeutic Innovation Laboratory CNRS, Strasbourg Institute for Drug Discovery and Development (IMS) Strasbourg University Strasbourg France; ^10^ Department of Pharmaceutical Sciences Faculty of Pharmacy São Paulo University Ribeirão Preto Brazil; ^11^ Postgraduate Program in Pharmaceutical Science, Postgraduate Program in Oral Science Department of Biophysical and Pharmacology Federal University of Rio Grande Norte Natal Brazil

**Keywords:** *Commiphora*, chemotherapy, 5‐fluorouracil, mucositis

## Abstract

This study evaluated the phytochemical composition, antimicrobial activity, and in vivo protective effects of the hydroethanolic extract from *Commiphora leptophloeos* stem bark (HECL) against 5‐fluorouracil (5‐FU)‐induced intestinal mucositis. Despite the extensive traditional use of *C. leptophloeos* in gastrointestinal disorders, its stem bark extract has not yet been chemically characterized nor systematically investigated in experimental models of chemotherapy‐induced intestinal injury. Phytochemical analysis by mass spectrometry identified 21 metabolites, mainly procyanidins and flavonoids. Antimicrobial screening against 90 bacterial strains showed selective inhibition of Gram‐positive bacteria at 40–80 µg/mL. In vivo, mucositis was induced in female Swiss mice with 5‐FU (450 mg/kg, i.p.). Treatment with HECL (10 mg/kg) significantly preserved villus and crypt architecture (*p* < 0.05) and reduced IL‐6 levels (*p* < 0.05). HECL also increased MUC‐2 expression (*p* < 0.05), while maintaining intestinal barrier integrity, as indicated by occludin immunostaining. Additionally, the extract modulated oxidative stress (malondialdehyde) and acetylcholinesterase activity. Overall, HECL demonstrated bioactive potential with broad‐spectrum antimicrobial activity and protective effects against chemotherapy‐induced intestinal injury, supporting its role as a therapeutic adjuvant in intestinal disorders.

## Graphical abstract

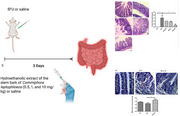

## 1 Introduction

Medicinal plants have played a fundamental role in traditional and modern pharmacotherapy [1, 2]. Ethnopharmacological knowledge has been especially valuable in guiding the selection of plant species with potential therapeutic applications in conditions characterized by inflammation, oxidative stress, and epithelial damage, which are commonly exacerbated by antineoplastic therapies [3, 4].

Chemotherapy remains a cornerstone of cancer treatment worldwide, and is routinely employed either alone or in combination with surgery, radiotherapy, and targeted therapies. Despite its effectiveness, chemotherapy is frequently associated with off‐target toxicity in rapidly proliferating normal tissues, reinforcing the need for safe adjuvant interventions capable of mitigating adverse effects. Among the different classes of chemotherapeutic agents, antimetabolites play a central role in the management of solid tumors, particularly those characterized by high proliferative rates. 5‐Fluorouracil (5‐FU) is one of the most widely used antimetabolites in clinical oncology and constitutes a first‐line or adjuvant therapy for several malignancies, including colorectal, breast, gastric, pancreatic, and head and neck cancers [5, 6, 7, 8]. Acting primarily through the inhibition of thymidylate synthase and the incorporation of fraudulent nucleotides into DNA and RNA, 5‐FU effectively suppresses tumor cell proliferation and induces apoptosis [9]. Despite its proven efficacy and long‐standing clinical use, the nonspecific cytotoxic action of 5‐FU also affects rapidly dividing normal tissues, particularly the gastrointestinal epithelium, leading to dose‐limiting toxicities that compromise treatment adherence and patient quality of life [5].

The pathogenesis of chemotherapy‐induced intestinal mucositis (IM) is a complex, dynamic, and multistep process that has been extensively characterized by the biological model proposed by Sonis. According to this model, mucositis develops through five overlapping phases that involve direct cytotoxic injury, oxidative stress, inflammatory signaling, tissue breakdown, and subsequent repair [5, 10, 11].

The initiation phase occurs immediately after exposure to chemotherapeutic agents such as 5‐fluorouracil and is characterized by direct DNA damage in rapidly dividing intestinal crypt cells, along with excessive generation of reactive oxygen species (ROS). These early events disrupt cellular homeostasis, induce mitochondrial dysfunction, and act as primary triggers for downstream inflammatory cascades [5].

In the upregulation and message generation phase, oxidative stress and cellular injury activate key transcription factors, particularly nuclear factor kappa B (NF‐κB). This activation leads to increased expression of proinflammatory mediators, including tumor necrosis factor‐α (TNF‐α), interleukin‐1β (IL‐1β), and interleukin‐6 (IL‐6), which further impair epithelial renewal and promote apoptosis of intestinal cells [5, 10].

The signal amplification phase is driven by positive feedback mechanisms in which cytokines, chemokines, and matrix metalloproteinases intensify tissue damage. Continued ROS production and sustained inflammatory signaling exacerbate crypt loss, compromise epithelial integrity, and amplify mucosal injury beyond the initial cytotoxic insult [10].

The ulceration phase represents the most clinically severe stage of IM and is characterized by extensive epithelial denudation, disruption of the intestinal barrier, and exposure of the underlying lamina propria. This phase is associated with severe gastrointestinal symptoms, including diarrhea, abdominal pain, malabsorption, and increased risk of bacterial translocation and systemic infection [5, 10, 11].

Finally, the healing phase involves epithelial restitution through proliferation, differentiation, and migration of surviving intestinal stem cells, leading to gradual restoration of mucosal structure and function once chemotherapy pressure is relieved. However, incomplete or delayed healing may predispose patients to persistent inflammation and long‐term gastrointestinal dysfunction [10, 11].

Within this biological framework, therapeutic strategies capable of exerting dual actions—attenuating oxidative and inflammatory damage during the early phases while supporting tissue repair and functional recovery during later stages—are particularly attractive for the management of chemotherapy‐induced intestinal mucositis.

Within the field of ethnopharmacology, *Commiphora leptophloeos* has attracted increasing interest due to its traditional use in inflammatory conditions and wound healing. Despite empirical evidence supporting its medicinal applications, scientific investigations into its biological properties and mechanisms of action remain limited. Studies involving other species of the Commiphora genus report anti‐inflammatory, antioxidant, and tissue‐repair activities, reinforcing the rationale for further investigation of *C. leptophloeos* [12, 13, 14, 15]. *C. leptophloeos* (Mart.) J.B. Gillett (Burseraceae) emerges as a relevant medicinal species native to Brazil, traditionally used by indigenous and rural communities for the treatment of wounds, inflammatory processes, infections, and gastrointestinal disorders [16, 17, 18, 19, 20, 21]. In a recent study, Da Silva et al. (2024) investigated the phytochemical profile and therapeutic effects of *C*. leptophloeos leaf extract in the context of inflammatory bowel disease (IBD). The extract, characterized by high levels of phenolic acids and flavonoids, exhibited anti‐inflammatory properties by decreasing proinflammatory mediators both in activated macrophages and in a 2,4‐dinitrobenzenesulfonic acid (DNBS)‐induced colitis model in mice. Intragastric administration of the extract at doses of 300, 400, and 500 mg/kg resulted in the suppression of key inflammatory signaling pathways. These results point to the potential of *C*. leptophloeos as a candidate for IBD therapy and support the need for further investigation [15].

Although previous studies have demonstrated antibacterial and anti‐inflammatory properties of *C. leptophloeos*, most investigations focused on the leaf extract and tested only a limited number of microbial strains [19]. To date, the potential of the stem bark extract has not been systematically evaluated in the context of chemotherapy‐induced intestinal mucositis. This study therefore addresses this gap by characterizing the phytochemical composition, assessing antibacterial activity against a broad panel of clinical isolates, and investigating the protective effects of the stem bark extract in a 5‐FU‐induced mucositis model.

Unlike the leaf extract, the stem bark contains higher levels of proanthocyanidins and glycosylated flavonoids, compounds strongly associated with antibacterial, antioxidant, and mucosal‐protective properties [22, 23, 24]. Therefore, investigating the stem bark is consistent with ethnopharmacological practices and may reveal unique or complementary bioactive activities compared with the leaves (Table 1).

**TABLE 1:** Histopathological grading scores.

Scores	Description of the findings
0	Normal architecture of villi and crypts; absence of inflammatory changes.
1	Mild alterations including villus shortening and partial loss of crypt architecture; slight inflammatory infiltrate; discrete vacuolization and mucosal edema; muscle layer preserved.
2	Marked villus blunting with epithelial flattening and vacuolization; focal crypt necrosis; moderate inflammatory infiltrate; edema and vacuolization in both mucosa and submucosa; muscle layer remains intact.
3	Severe villus atrophy and extensive crypt necrosis; intense inflammatory infiltration; prominent edema and vacuolization in mucosal, submucosal, and muscular layers; presence of neutrophils in the muscularis layer.

Based on this background, we hypothesized that the hydroethanolic extract of *C. leptophloeos* stem bark exerts beneficial effects on chemotherapy‐induced intestinal injury by modulating inflammation, oxidative stress, epithelial integrity, and microbial susceptibility. Thus, the present study aimed to characterize the phytochemical composition of the stem bark extract, assess its antibacterial activity against an extensive panel of bacterial isolates, and investigate its effects on intestinal architecture, inflammatory markers, oxidative stress, and barrier function in a 5‐fluorouracil–induced mucositis model in mice.

## 2 Results and Discussion

### 2.1 Phytochemical Putative Annotation of *Commiphora Leptophloeos* Extract

**FIGURE 1:**
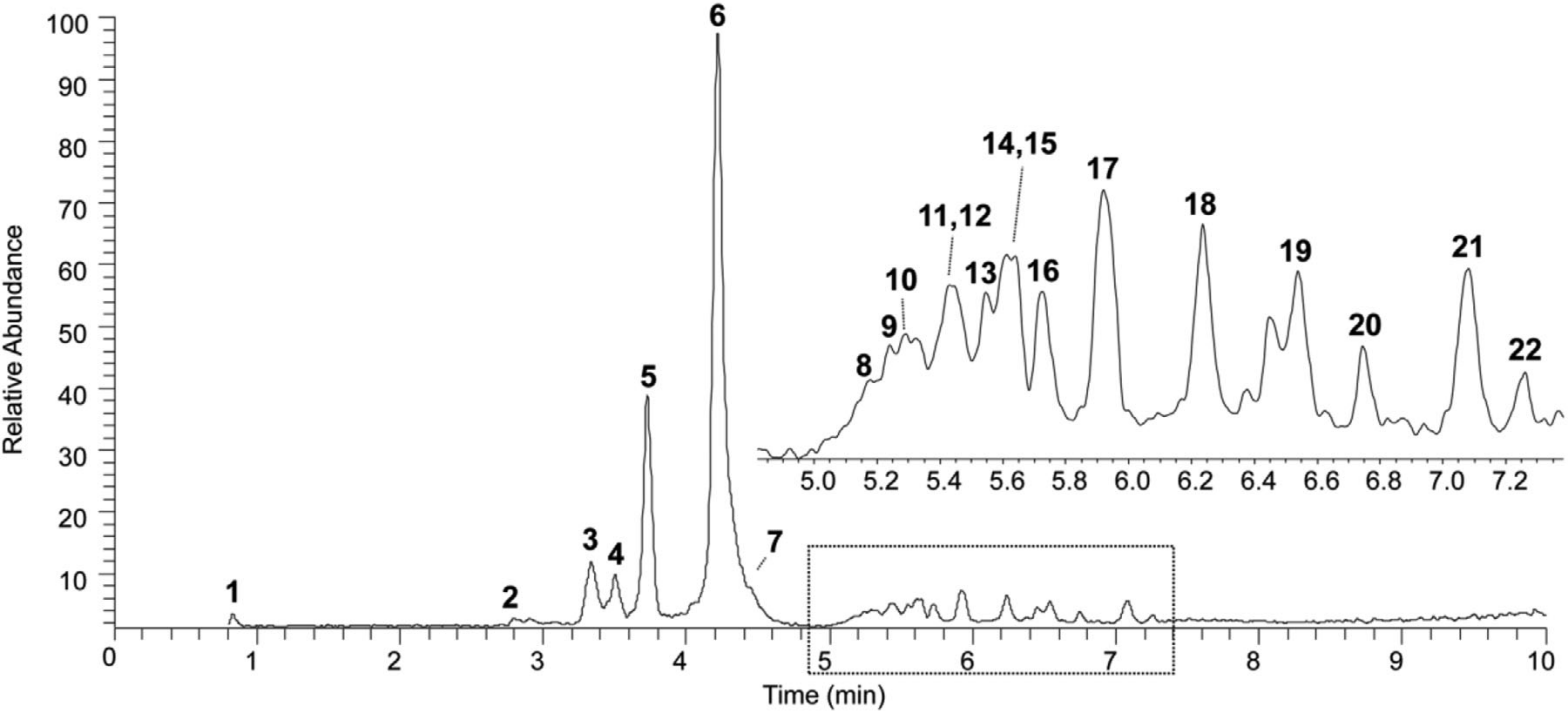
Base peak chromatogram (BPC) obtained by UPLC‐ESI‐IT‐MS analysis in negative ion mode of the HECL stem bark. Peaks correspond to compounds tentatively annotated based on MS/MS fragmentation patterns and comparison with literature data.

This approach resulted in the putative annotationannotation of 21 compounds at metabolomics standards initiative (MSI) confidence level 3 [25] (Figure 1, Table 2), based on their [M–H]^−^ ions, MS/MS fragmentation patterns, and comparison with previously reported metabolites in the *Commiphora* genus or species. Although 22 peaks were detected in the chromatogram, one peak could not be structurally characterized and therefore remains unidentified. In general, the fragmentation profiles were primarily characterized by neutral losses of hexose (–162 Da), deoxyhexose (–146 Da), and galloyl (–152 Da) units, in addition to *retro‐Diels–Alder* and heterocyclic ring fission cleavages typical of proanthocyanidins and flavonoids, as already reported in our previous studies.

**TABLE 2:** Compounds tentatively identified in *C. leptophloeos* stem bark extract (HECL) by mass spectrometry.

Peak	Rt (min)	[M‐H]^−^ *m/z*	MS/MS *m/z*	Tentative identification
1	0.83	191	173, 127, 85	Quinic acid
2	2.80	879	577, 451, 425, 289	B‐type trimeric proanthocyanidin
3	3.33	865	577, 451, 425, 289	B‐type trimeric procyanidin
4	3.50	1153	865, 577, 451, 425, 289	B‐type tetrameric procyanidin
5	3.72	577	429, 357, 327, 285	B‐type dimeric procyanidin
6	4.22	865	577, 451, 425, 289	B‐type trimeric procyanidin
7	4.45	1153	865, 577, 451, 425, 289	B‐type tetrameric procyanidin
8	5.17	1153	865, 577, 451, 425, 289	B‐type tetrameric procyanidin
9	5.24	1153	865, 577, 451, 425, 289	B‐type tetrameric procyanidin
10	5.29	1441	1153, 865, 577, 289	B‐type pentameric procyanidin
11	5.43	863	575, 451, 425, 289	A‐type trimeric proanthocyanidin
12	5.49	1151	863, 577, 451, 425, 289	A‐type tetrameric proanthocyanidin
13	5.55	447	357, 327, 285	Luteolin‐hexose
14	5.61	447	429, 357, 327, 285	Luteolin‐hexose
15	5.61	599	447, 429, 357, 327, 285	Galloylorientin
16	5.72	865	577, 451, 425, 289	B‐type trimeric procyanidin
17	5.92	431	341, 311, 283	Apigenin‐hexose
18	6.23	431	413, 341, 311, 283	Apigenin‐hexose
19	6.54	501	301	Ellagic acid hexoside
20	6.74	535	—	Not identified
21	7.08	447	301, 179, 151	Quercetin‐deoxyhexose
22	7.26	623	477, 461, 301	Metoxyquercetin‐hexose‐deoxyhexose

References of the studies listed in Table 2. Peaks: 1–6 [26]; 7–12^18^, 13–18 [27], 19 [28]; 21 and 22 [27].

Thus, the first compound detected (peak 1, *m*/*z* 191) was tentatively identified as quinic acid, which fragmented into *m*/*z* 173, *m*/*z* 127, and *m*/*z* 85 [16]. The main peaks from 2 to 10 exhibited precursor ions at *m*/*z* 577, *m*/*z* 865, *m*/*z* 879, and *m*/*z* 1153 corresponding to the dimer, trimer, and tetramer procyanidins with type B bond and their respective isomers. These oligomeric structures indicate a high degree of proanthocyanidin polymerization, consistent with previously reported profiles for this species [17, 29]. Two A‐type procyanidins were also tentatively identified. The trimer (peak 11, *m*/*z* 863) showed a fragment at *m*/*z* 575 (–288 and –2 Da), indicative of an additional ether bond linking the flavan‐3‐ol units, a hallmark of A‐type linkages [30]. The tetrameric A‐type proanthocyanidin (peak 12, *m*/*z* 1151) yielded fragment ions at *m*/*z* 863, 577, 451, 425, and 289, confirming a similar interflavan connection.

Additionally, several flavonoid glycosides were also annotated. Peaks 13 and 14 (*m*/*z* 447) were attributed to luteolin‐hexoside and their respective isomers. Their MS/MS spectra showed fragmentation patterns with ions at *m*/*z* 357, 327, and 285 for compound 13, and *m*/*z* 429, 357, 327, and 285 for compound 14, consistent with cross‐ring cleavages of bound hexose moieties [15]. Peak 15 (*m*/*z* 599) was tentatively identified as galloylorientin, displaying a primary loss of the galloyl group (−152 Da), followed by sugar cleavage, and resulting in *m*/*z* 285 (luteolin aglycone). Peaks 17 and 18 (*m*/*z* 431) were annotated as isomer of apigenin‐hexoside, with fragment ions at *m*/*z* 341, 311, and 283 for compound 17, and *m*/*z* 413, 341, 311, and 283 for compound 18. These fragmentation patterns support the annotation of glycosylated flavones [15].

Added to this, a compound at *m*/*z* 501 (peak 19) was proposed as ellagic acid hexoside. Its fragmentation led to a single dominant ion at *m*/*z* 301, representing the ellagic acid aglycone after the loss of a hexose moiety (–162 Da). Although it is not possible to annotate the compound at *m*/*z* 535 (peak 20) ​​due to its low intensity and absence of informative MS/MS fragmentation. Peak 21 (*m*/*z* 447), in contrast, was annotated as quercetin‐deoxyhexose, showing characteristic fragment ions at *m*/*z* 301 (quercetin aglycone), 179, and 151. Finally, peak 22 (*m*/*z* 623) was tentatively identified as methoxyquercetin‐hexose‐deoxyhexose, fragmenting sequentially to *m*/*z* 477, 461, and ultimately *m*/*z* 301, confirming the presence of a diglycosylated methoxylated flavonol [15].

It is important to emphasize that the purpose of the present MS‐based analysis was to confirm the conservation of the phytochemical profile of the hydroethanolic stem bark extract rather than to provide definitive structural identification. Several of the major constituents of HECL, particularly B‐type procyanidins, had been previously isolated from this species and their structures unequivocally elucidated by NMR and mass spectrometry [17, 18]. Moreover, these compounds were previously quantified using a validated HPLC–UV–ELSD method, which demonstrated that B‐type dimeric procyanidin is the major constituent of HECL and a strong candidate as a chemical marker for quality control. B‐type trimeric, tetrameric, and pentameric procyanidins were also quantified, although they occur at lower concentrations compared to the dimer [6]. Therefore, the present results reinforce the predominance of procyanidin derivatives in HECL and confirm the stability of its characteristic phytochemical pattern.

### 2.2 Antibacterial Activity of HECL

The HECL exhibited interesting antibacterial activity against several Gram‐positive clinical and reference strains. The inhibition was observed for *Staphylococcus aureus, Staphylococcus epidermidis, Staphylococcus intermedius, Staphylococcus lugdunensis, Staphylococcus saprophyticus, Staphylococcus haemolyticus, Enterococcus faecalis, Enterococcus avium, Corynebacterium minutissimum, Corynebacterium striatum, and Bacillus cereus*, MIC ranging from 40 to 100 µg/mL, as detailed in Table 3.

**TABLE 3:** Minimum inhibitory concentration (MIC) of the HECL and the reference antibiotic ofloxacin against Gram‐positive bacteria. All assays were conducted in triplicate (*n* = 3).

Bacteria specie	Reference	MIC (µg/mL)	
		Ofloxacin	HECL
*Bacillus cereus*	CIP 6624	0.25	60
*Bacillus cereus*	N551	0.25	60
*Bacillus cereus*	N839	0.25	40
*Bacillus cereus*	N1060	0.5	40
*Bacillus cereus*	N1139	0.5	40
*Corynebacterium minutissimum*	CIP 100652T	2	40
*Corynebacterium striatum*	N1129	0.5	60
*Enterococcus avium*	CIP 104 053	0.5	> 100
*Enterococcus avium*	N1061	2	> 100
*Enterococcus avium*	N1064	2	> 100
*Enterococcus casseliflavus*	CIP 103.018T	4	> 100
*Enterococcus casseliflavus*	N850	2	> 100
*Enterococcus durans*	CIP 104 999	2	> 100
*Enterococcus durans*	N569	1	> 100
*Enterococcus durans*	N1136	1	> 100
*Enterococcus faecalis*	CIP103.214	0.5	100
*Enterococcus faecalis*	CIP 104 676	0.5	100
*Enterococcus faecalis*	N491	2	80
*Enterococcus faecalis*	N571	2	100
*Enterococcus faecalis*	N650	1	100
*Enterococcus faecalis*	N755	> 128	> 100
*Enterococcus faecium*	CIP 103.014	87	> 100
*Enterococcus faecium*	CIP 107.387	16	> 100
*Enterococcus faecium*	N490	2	> 100
*Enterococcus faecium*	N734	> 128	> 100
*Enterococcus faecium*	N838	> 128	> 100
*Enterococcus faecium*	N853	> 128	> 100
*Enterococcus gallinarum*	CIP 105 985	2	> 100
*Enterococcus gallinarum*	N743	2	> 100
*Enterococcus hirae*	CIP 5855	2	> 100
*Listeria monocytogenes*	CIP 103.575	2	> 100
*Listeria monocytogenes*	N783	2	> 100
*Listeria monocytogenes*	N836	2	> 100
*Listeria monocytogenes*	N851	2	> 100
*Listeria monocytogenes*	N884	2	> 100
*Staphylococcus aureus*	ATCC 9144	0.25	100
*Staphylococcus aureus*	ATCC 6538P	0.25	60
*Staphylococcus aureus*	ATCC 25923	0.5	60
*Staphylococcus aureus*	ATCC 29213	0.5	60
*Staphylococcus aureus*	CIP 57.10	0,25	100
*Staphylococcus aureus*	CRBI 21.21	64	40
*Staphylococcus aureus*	E372	16	60
*Staphylococcus aureus*	E373	1	80
*Staphylococcus aureus*	E378	4	60
*Staphylococcus aureus*	E410	0.5	80
*Staphylococcus aureus*	E415	0.5	80
*Staphylococcus epidermidis*	CIP 53.124	0.5	100
*Staphylococcus haemolyticus*	CIP 81.56	0.25	> 100
*Staphylococcus intermedius*	N987	32	60
*Staphylococcus lugdunensis*	ATCC 43.809	1	100
*Staphylococcus saprophyticus*	CIP 76125	1	> 100

In contrast, no antibacterial activity was observed against Gram‐negative strains at concentrations up to 100 µg/mL [57], indicating a selective effect of the HECL predominantly toward Gram‐positive bacteria. This selective activity suggests a potential application of HECL against Gram‐positive pathogens involved in gastrointestinal infections.

### 2.3 Deaths, Body Weight, Peripheral Blood Leucocyte Counts, and Hematological Analysis

**FIGURE 2:**
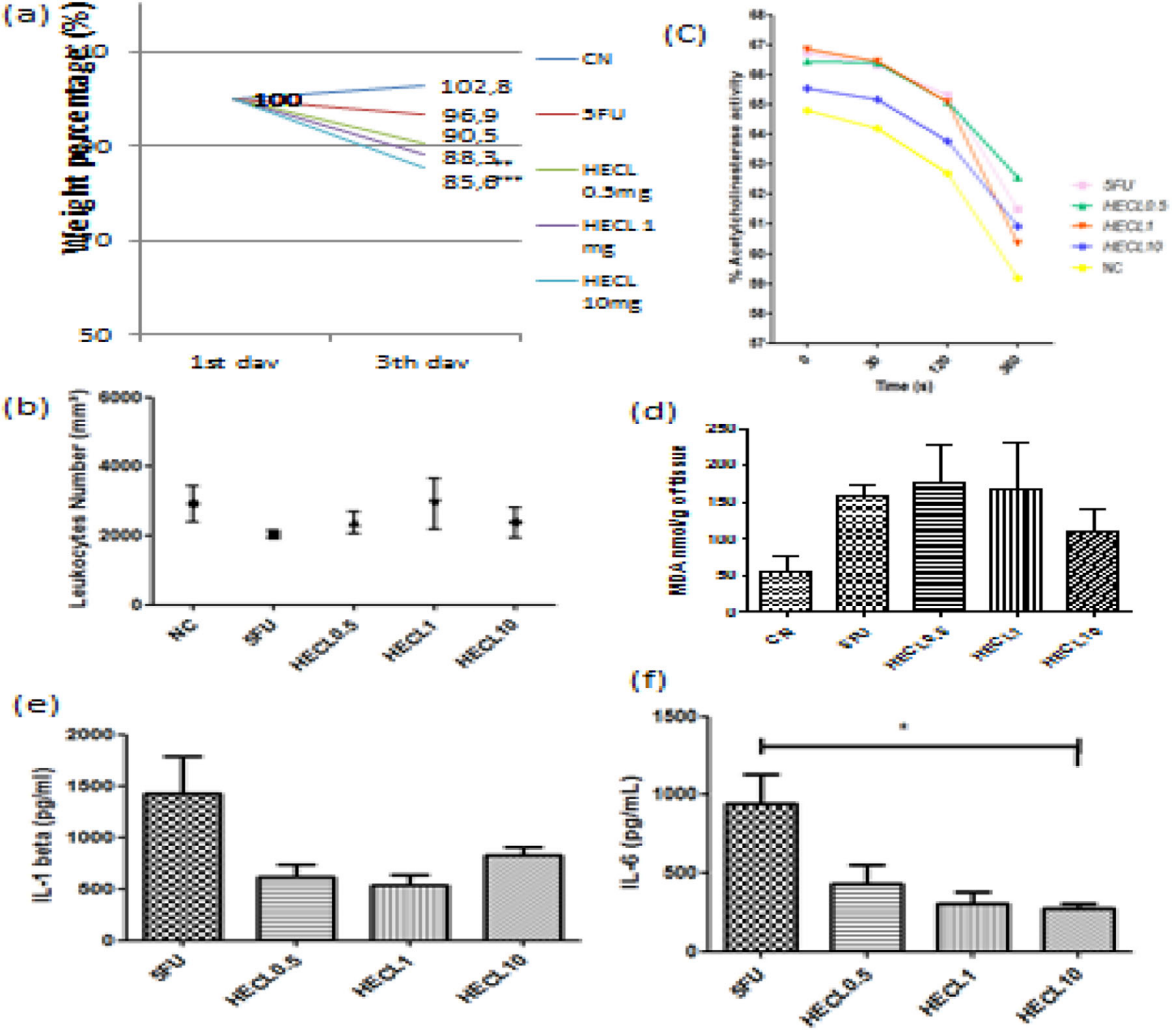
Effect of treatments on different parameters in experimental animals (a): Body weight percentage on the first and third days; (b) total leukocytes count; (c) percentage of acetylcholinesterase activity at different time points (seconds); (d) malonaldehyde (MDA) levels expressed as nmol/g of tissue. Levels of proinflammatory (e) cytokines IL‐1β (left) and (f) IL‐6 (right) in intestinal tissue of mice treated with 5‐fluorouracil (5FU) and hydroethanolic extract of *Commiphora leptophloeos* (HECL) at doses of 0.5, 1, and 10 mg/kg. Data are expressed as mean ± SEM. A significant reduction in IL‐6 levels was observed in HECL‐10 group compared to the 5FU group Groups: CN (negative control);5FU; HECL0.5; HECL1 e HECL10. *p < 0.05, **p < 0.01;*** *p* < 0.001.

During the experiments, no animal deaths were observed in the NC, 5‐FU, and HECL‐treated groups for three days. Regarding body weight, the NC group showed an increase in weight percentage (102.8%) on day 3, whereas the 5‐FU group experienced weight loss (96.9%). HECL treatment did not prevent the weight loss induced by 5‐FU, although animals in the 1 and 10 mg/kg groups exhibited significant weight recovery compared to the positive control group (*p* < 0.01 and *p* < 0.001, respectively), (Figure 2a). In HECL 0.5 mg/kg, body weight percentage was 90.5 %, *p* > 0.05.

The global leukocyte count data showed values of 2920 ± 1189/mm^3^ for the NC group and 2028 ± 217.5/mm^3^ for the 5‐FU group. For the HECL‐treated groups at doses of 0.5, 1, and 10 mg, the leukocyte counts were 2380 ± 712.9/mm^3^, 2920 ± 1622/mm^3^, and 2386 ± 975.6/mm^3^, respectively (Figure 2b).

Table 4 presents the reference values and biochemical findings for ALT/TGP (U/L), creatinine (mg/dL), and urea (mg/dL), which remained within the reference range.

**TABLE 4:** Biochemical parameters of hepatic and renal function.

Groups	ALT (U/L)	Creatinine (mg/dL)	Urea (mg/dL)
NC	58.14 ± 23.54	0.38 ± 0.19	44.2 ± 8.41
5FU	56.30 ± 25.89	0.4 ± 0.18	29.6 ± 3.13
HECL 0.5	49.80 ± 29.55	0.392 ± 0.07	55.40 ± 5.68(a)
HECL 1	48.78 ± 40.58	0.292 ± 0.11	43.40 ± 10.64
HECL 10	70.32 ± 45.39	0.408 ± 0.04	36.20 ± 7.26
Reference values ​​(minimum and maximum)	24–131	0.1–0.62	45–90

NC: Negative control; 5FU (5 Fluorouracil group); Hydroethanolic extract from *C. leptophloeos* stem bark (HECL) doses: 0.5, 1, and 10 mg/kg. (a) 5FU vs HECL 0.5 < 0.01. Reference values ​​ [31].

In all groups, ALT/TGP values were within the reference range (24–131 U/L), indicating no hepatic injury induced by treatments. The NC (58.14 ± 23.54 U/L), 5FU (56.30 ± 25.89 U/L), and HECL‐treated groups (49.80 ± 29.55; 48.78 ± 40.58, and 70.32 ± 45.39 U/L for 0.5, 1, and 10 mg/kg, respectively) did not show significant deviations from reference values.

Creatinine levels in all groups also remained within the normal range (0.1–0.62 mg/dL), suggesting preserved renal function. The NC group presented 0.38 ± 0.19 mg/dL, while the 5FU group had 0.4 ± 0.18 mg/dL. HECL groups showed similar values (0.392 ± 0.07; 0.292 ± 0.11; 0.408 ± 0.04 mg/dL for 0.5, 1, and 10 mg/kg, respectively).

Regarding urea levels (reference 45–90 mg/dL), most groups remained within the normal range except for the 5FU group, which presented a lower mean value (29.6 ± 3.13 mg/dL) and significantly statistical compared with HECL 0.5 mg/kg group (*p* < 0.01). The NC, HECL 0.5 mg/kg, HECL 1 mg/kg, and HECL 10 mg/kg groups showed urea values within the reference range (44.2 ± 8.41; 55.40 ± 5.68 mg/dL; 43.40 ± 10.64; and 36.20 ± 7.26 mg/dL, respectively).

### 2.4 Malonaldehyde Dosage (MDA)

Malondialdehyde production results indicated a trend toward reduced levels in the HECL 10 mg‐treated group (110.3 ± 57.4), suggesting a potential therapeutic effect. Although not statistically significant (*p* > 0.05), MDA levels showed a downward trend in the HECL 10 mg group, suggesting a possible reduction in lipid peroxidation, (Figure 2d).

### 2.5 Acetylcholinesterase Activity and Fecal Retention in the Intestine

Our findings showed that for the CN and HECL10 groups, score 0 with absence of stool retention along the intestine, while retention was intense in volume and extension for the 5FU, HECL0.5, and HECL1 groups.

Acetylcholinesterase (AChE) activity was evaluated in all experimental groups at different time points (0, 30, 120, and 360 s). As shown in Figure 2c, the NC group displayed the lowest percentage of AChE activity across the time course, reflecting physiological conditions and normal intestinal motility. In contrast, the 5FU group maintained relatively high AChE activity values, with only a modest decrease over time. Among the HECL‐treated groups, the activity pattern varied with the dose: HECL 0.5 mg and HECL 1 mg showed intermediate AChE activity levels, closer to those of the 5FU group at the early time points, but with a progressive decline after 120 and 360 s. HECL 10 mg exhibited a profile more similar to NC, demonstrating a marked decrease in AChE activity by the end of the observation period. Overall, the HECL treatments, particularly at higher doses, produced AChE activity patterns approaching those of the NC group, suggesting a potential modulatory effect on enzyme activity compared with 5FU treatment.

### 2.6 Cytokine Levels (IL‐1β and IL‐6)

Treatment with *C. leptophloeos* bark extract (HECL) significantly attenuated IL‐1β (pg/mL). According to nonparametric Mann–Whitney analysis followed by Dunn's post hoc test, animals treated with HECL at 1 mg/kg showed a statistically significant reduction in IL‐1β concentrations compared to the 5‐FU group (*p* < 0.05), Figure 2e. Treatment with *C. leptophloeos* bark extract (HECL) significantly attenuated IL‐6 levels. According to nonparametric Mann–Whitney analysis followed by Dunn's post hoc test, animals treated with HECL at 1 and 10 mg/kg showed a statistically significant reduction in IL‐6 concentrations compared to the 5‐FU group (*p* < 0.05). Among the tested doses, HECL treatment resulted in a marked suppression of IL‐6 release, with cytokine levels approaching those observed under noninflamed conditions, Figure 2f.

### 2.7 Histopatological Analysis

**FIGURE 3:**
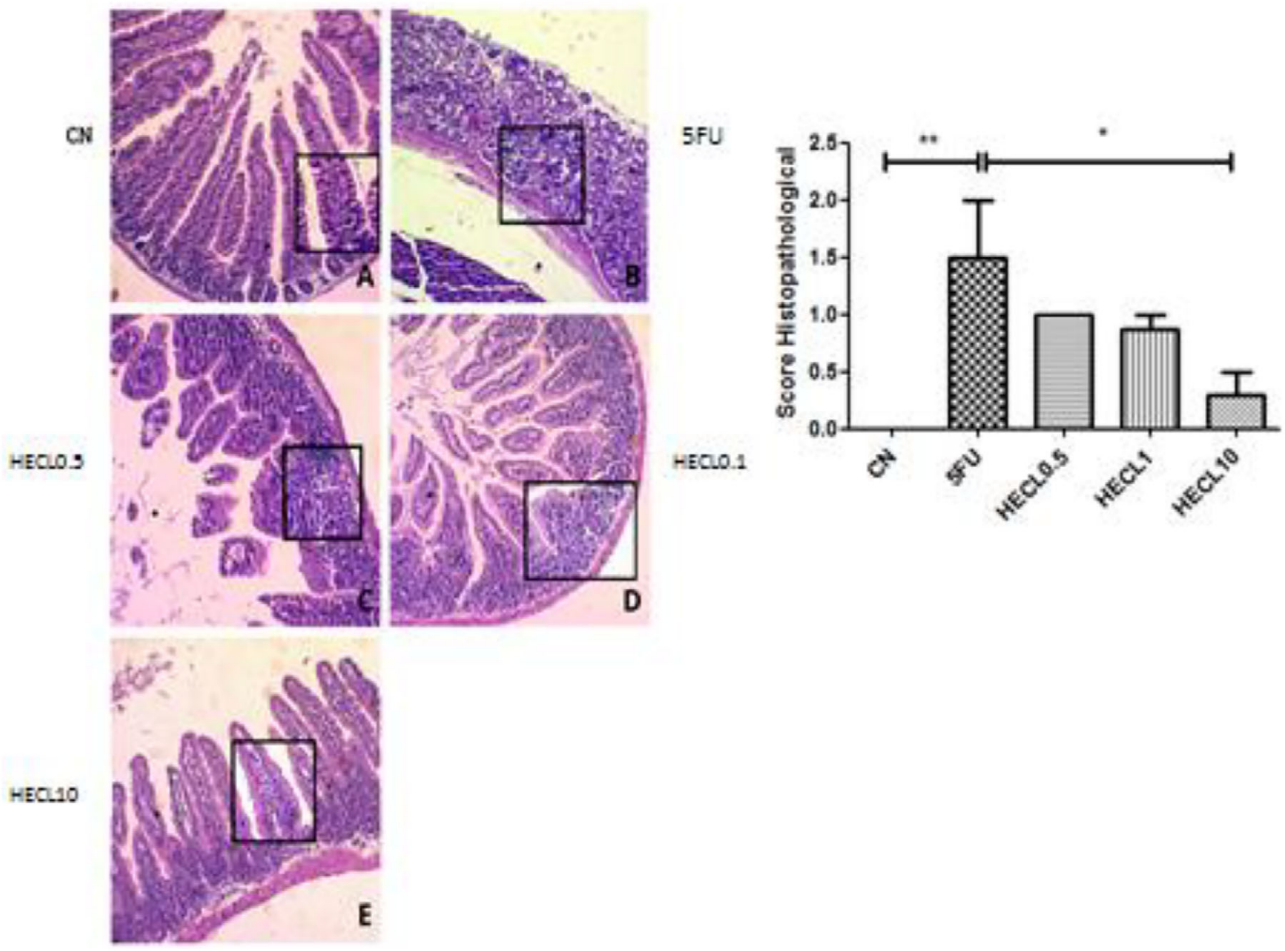
Histopathological analysis of duodenum tissues obtained from animals in control and treated groups. (A) Negative control group (CN) showing preservation of villi architecture and overall organ structure. (B) Positive control group (CP) demonstrating a marked loss of organ architecture, with villi shortening, loss of crypt architecture, presence of necrosis, vacuolization and edema in the muscular layer. (C) and (D) Treated groups CL 0.5 and CL 1 mg/kg, respectively, showing villi shortening, loss of crypt architecture, and sparse infiltration of inflammatory cells. (E) Group CL 10 mg/kg demonstrating preservation of intestinal villi and organ architecture, with absence of inflammatory cells. A –Negative control (NC); B: 5FU; C: HECL0.5; D: HECL1; E: HECL10. ** *p* < 0.01; * *p* < 0.05. Histological scores are expressed as median with interquartile range (IQR). Error bars represent the 25th–75th percentiles. Statistical analysis was performed using Kruskal–Wallis test followed by Dunn's post hoc test.

**FIGURE 4:**
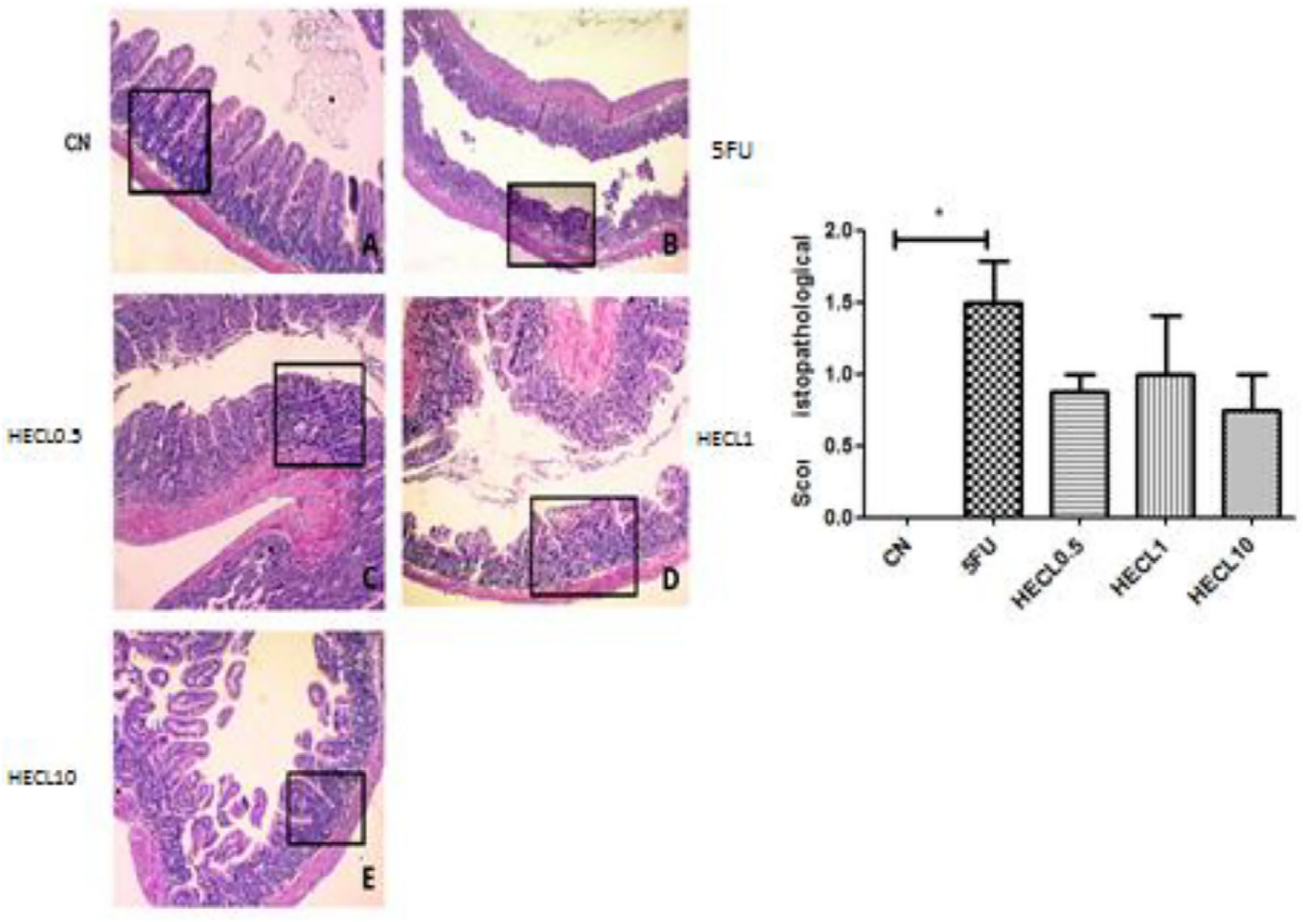
Histopathological analysis of ileum tissue obtained from animals in the control and treated groups. The negative control group (A) shows preservation of villi architecture, crypts, and organ structure. The positive control group (B) exhibits a significant loss of tissue architecture, including villi shortening, crypt disorganization, and necrosis. In the treated groups, CL 0.5 (C) and CL 1 (D), villi shortening, crypt disorganization, and sparse infiltration of inflammatory cells are observed. In the CL 10 group (E), greater preservation of intestinal villi and absence of inflammatory cells is evident. The bar graph on the right represents the histopathological score, where asterisks indicate a statistically significant difference compared to the negative control group. A –Negative control (NC); B: 5FU; C: HECL0.5; D: HECL1; E: HECL10. * *p* < 0.05. Histological scores are expressed as median with interquartile range (IQR). Error bars represent the 25th–75th percentiles. Statistical analysis was performed using Kruskal–Wallis test followed by Dunn's post hoc test.

Our findings revealed that 5‐FU induced intestinal damage across duodenum 1 (1.0–2.5) and Ileum 1.5 (1.0–2.5) intestinal segments (Figures 3 and 4) compared with CN 0.0 (0.0–0.0), *p* < 0.01 and *p* < 0.5, respectively. This damage manifested as shortened villi, compromised crypt architecture, submucosal edema, and a pronounced influx of inflammatory cells into the propria lamina.

**FIGURE 5:**
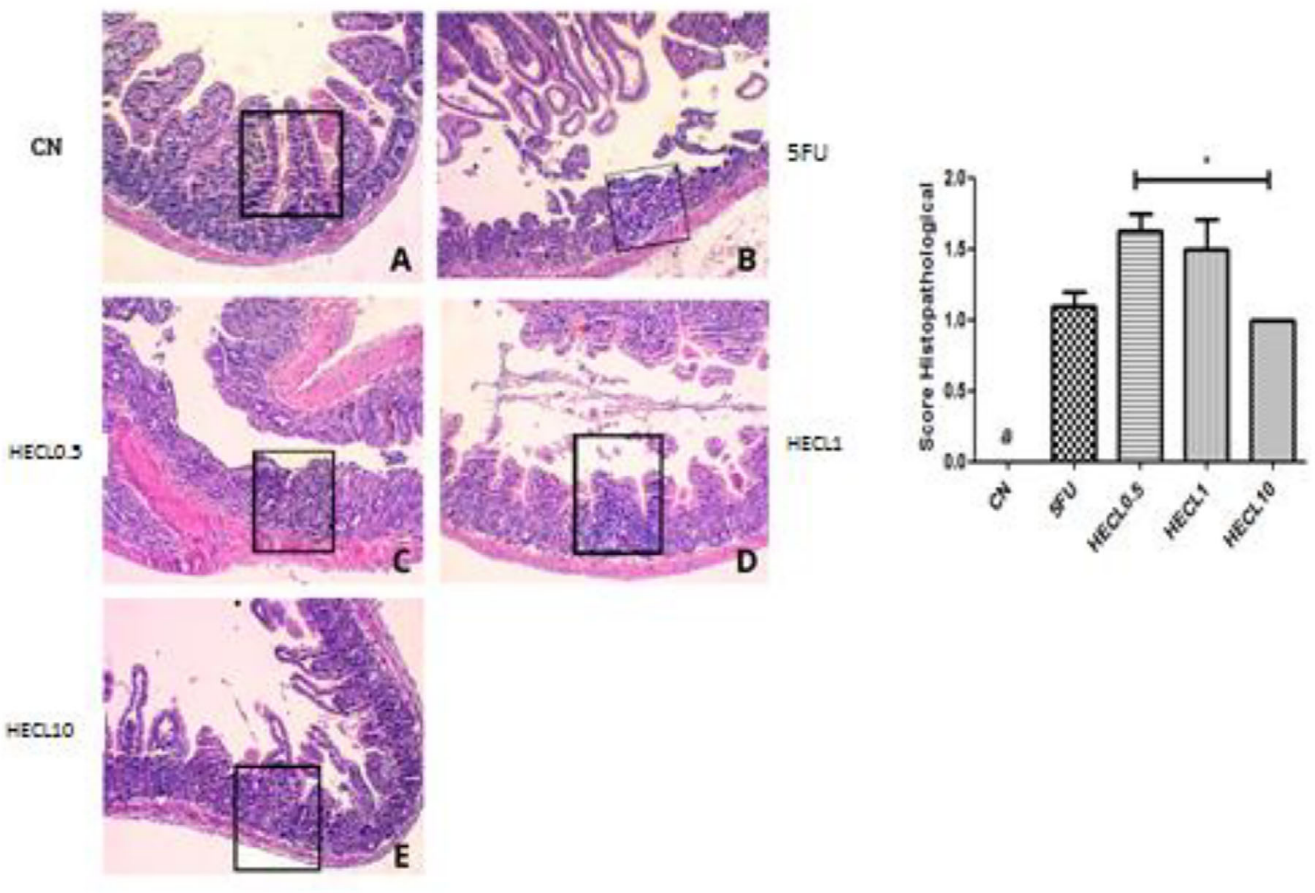
Histopathological analysis of jejune tissue obtained from control and treated groups: The negative control group (A) shows preservation of the villi architecture and overall organ structure. In the positive control group (B), there is a significant loss of organ architecture with villus shortening, crypt distortion, and necrosis. In the treated groups, CL 0.5 (C) and CL 1 (D), villus shortening, loss of crypt architecture, and sparse infiltration of inflammatory cells are observed. In group CL 10 (E), greater preservation of intestinal villi and sparse infiltration of inflammatory cells are seen. The bar graph on the right represents the histopathological score, where asterisks indicate a statistically significant. A –Negative control (NC); B: 5FU; C: HECL0.5; D: HECL1; E: HECL10. * *p* < 0.05. a = NC compared with all groups (p˂0.001). Histological scores are expressed as median with interquartile range (IQR). Error bars represent the 25th–75th percentiles. Statistical analysis was performed using Kruskal–Wallis test followed by Dunn's post hoc test.

HECL at 10 mg/kg significantly preserved the histological architecture of the duodenum, with scores 0 (0.0–0.75) close to those of the control group, indicating protection against 5‐FU‐induced mucosal injury (*p* < 0.05), Figure 3. Figure 5 showed jejunum with significantly difference between HECL0.5 score 1.5 (1.5–1.88) and HECL10, score 1.0 (1.0–1.0). The CN showed significantly difference compared all groups (p < 0.001).

**FIGURE 6:**
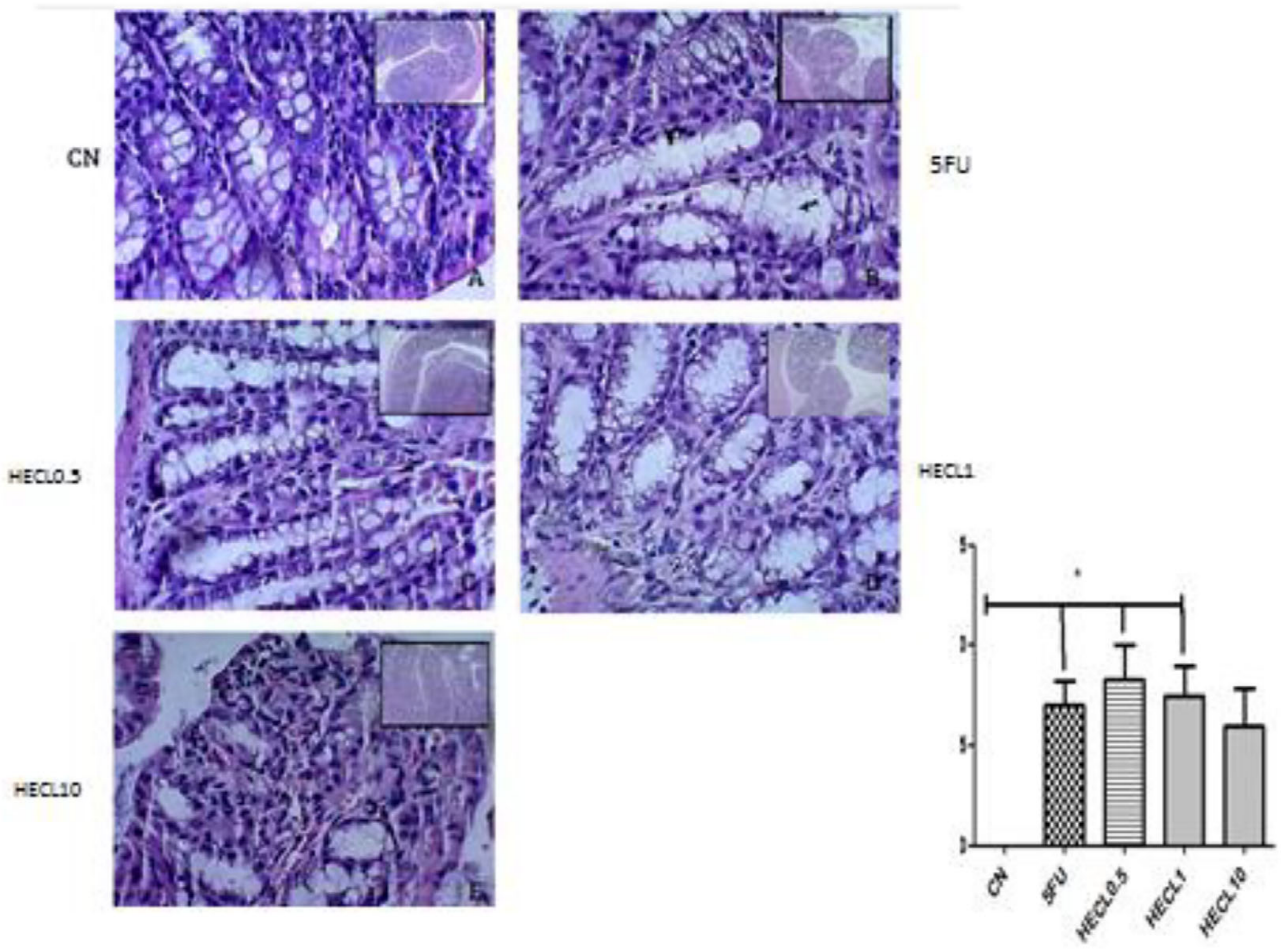
Representative histological sections of the colon (A–E) stained with hematoxylin and eosin (H&E). (A) Control group (CN) showing normal mucosal architecture with intact epithelial lining, preserved crypts, and abundant goblet cells. (B) 5‐FU group displaying marked epithelial disruption, shortening of crypts, inflammatory cell infiltration in the lamina propria, and reduction of goblet cells. (C) HECL0.5‐treated group with mostly preserved crypt structure, minimal inflammatory infiltration, and intact epithelial lining, indicating a protective effect. (D) HECL1‐treated group showing mild epithelial damage and slight crypt disruption with mild inflammatory infiltration. (E) HECL10‐treated group with nearly normal mucosal architecture, sparse inflammatory cells, and preserved goblet cells similar to control. Graph (C) depicts histopathological scores of the colon; the CN group shows a significant decresed compared with 5FU, HECL0.5 and HECL1.0 (*p < 0.05). A –Negative control (NC); B: 5FU; C: HECL0.5; D: HECL1; E: HECL10. Histological scores are expressed as median with interquartile range (IQR). Error bars represent the 25th–75th percentiles. Statistical analysis was performed using Kruskal–Wallis test followed by Dunn's post hoc test.

In Figure 6, a statistically significant difference was observed between the CN group score 0.0 (0.0–0.0) and the 5‐FU score 0.5 (0.5–1.0), HECL0.5 score 1.0 (0.5–1.0), and HECL1 score 0.75 (0.5–1.0) (*p* < 0.05). Notably, the HECL10 score 0.5 (0.25–1.0) did not show pathological morphological alterations in the colon region when compared to the CN group (*p* > 0.05).

### 2.8 MUC‐2 and Ocluddin Immunostaining

**FIGURE 7:**
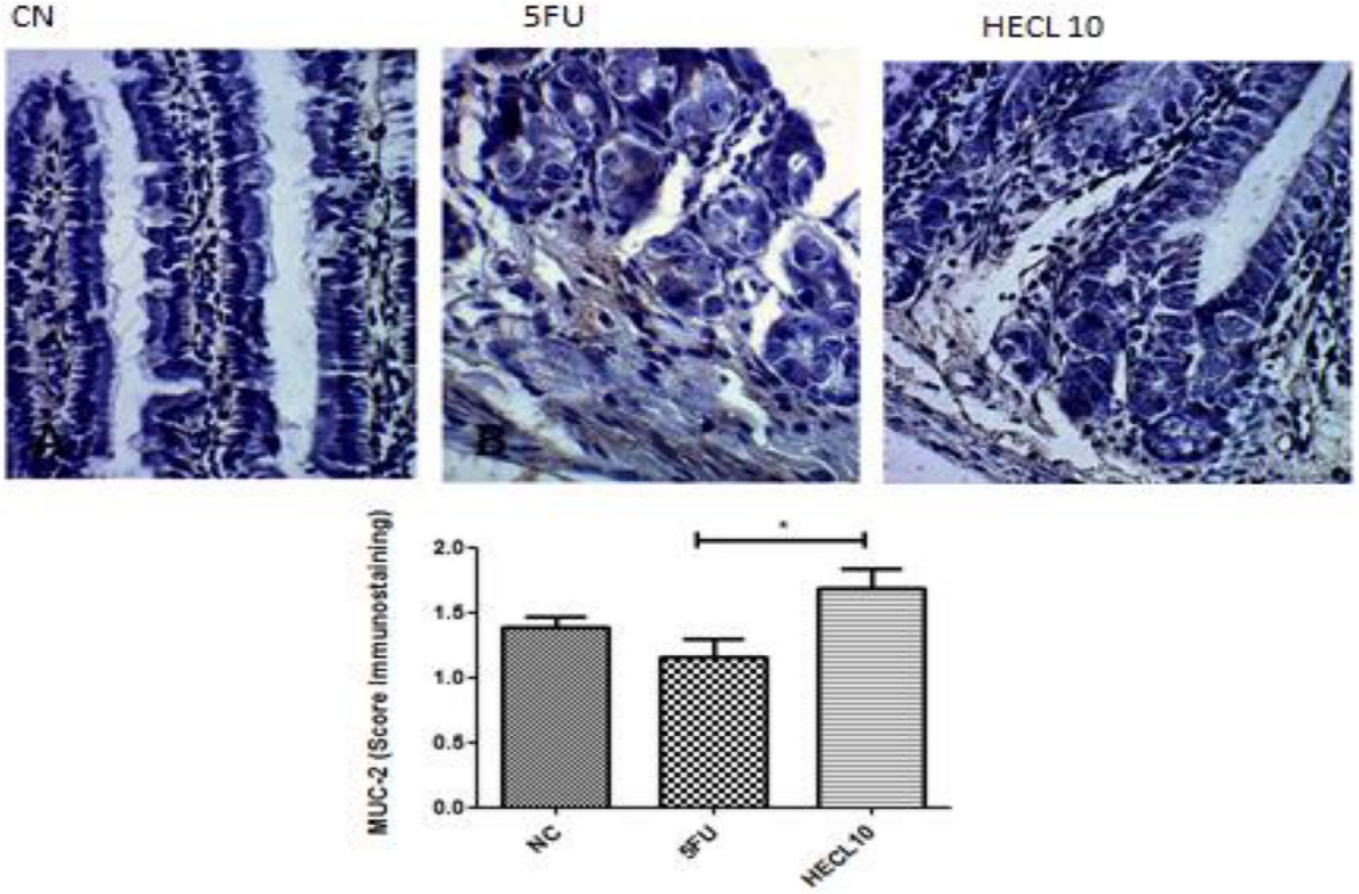
Immunohistochemical staining for MUC‐2 in colon tissue. (A) Control group (CN) showing strong and uniform MUC‐2 immunoreactivity along the mucosal epithelium and crypts, with intense staining of goblet cells. (B) 5‐FU‐treated group exhibiting marked reduction and focal loss of MUC‐2 immunostaining, with disruption of epithelial integrity and depletion of goblet cells. (C) HECL10‐treated group demonstrating enhanced MUC‐2 expression compared with the 5‐FU group, with increased staining intensity and restoration of mucosal architecture. The bar graph represents the quantitative MUC‐2 immunostaining score; the 5‐FU group shows a significant decrease compared to the control, whereas the HECL10 group presents significantly higher MUC‐2 expression (*p < 0.05), indicating mucosal protection by HECL10. Negative control (NC); 5FU; HECL10. Histological scores are expressed as median with interquartile range (IQR). Error bars represent the 25th–75th percentiles. Statistical analysis was performed using Kruskal–Wallis test followed by Dunn's post hoc test.

MUC‐2 expression in the duodenum was significantly increased in the HECL 10 mg/kg group [1.6 (1.4–2.0)] compared to the 5‐FU group [1.2 (0.8–1.6); *p* < 0.05] (Figure 7). In contrast, occludin expression did not significantly differ between groups (*p* > 0.05; no shown).

Previous phytochemical studies on various extracts from the stem bark of *C. leptophloeos*, including hexane, ethyl acetate, methanolic, ethanolic, and aqueous extracts, have developed a complex profile of bioactive compounds. Consistent with previous reports, HECL was rich in phenolic compounds, particularly oligomeric and polymeric proanthocyanidins, which are linked to its antimicrobial properties [19, 32]. Clearly, oligomeric and polymeric proanthocyanidins were tentatively identified as the main constituents, mainly related to the antifungal and antibacterial activities of the species [18, 20, 33, 34].

Mass spectrometry analysis of HECL in this study revealed that proanthocyanidins constitute the major class of secondary metabolites, as indicated by the higher intensity of peaks in the chromatographic profile. Additionally, glycosylated flavonoids were detected in the extract, albeit in smaller amounts. This finding is notable since such compounds are typically reported in leaf extracts rather than in stem bark, as demonstrated here. The presence of these glycosylated flavonoids may imply an enhanced pharmacological potential due to their recognized antioxidant and anti‐inflammatory activities [21, 34, 35].

HECL displayed antibacterial activity against a range of Gram‐positive bacteria, including *S. aureus*, *S. epidermidis*, *E. faecalis*, and *C. striatum*, pathogens commonly implicated in systemic infections in immunocompromised patients. Inhibition of these pathogens is particularly relevant due to their role in secondary infections associated with chemotherapy‐induced mucosal barrier disruption

The inhibition of *Bacillus cereus*, a causative agent of foodborne gastroenteritis, and *C. striatum*, an emerging multidrug‐resistant pathogen associated with bloodstream and gastrointestinal infections, further supports the potential utility of HECL in the treatment of secondary infections associated with mucosal barrier damage during intestinal mucositis. In contrast, no activity was observed against Gram‐negative bacteria at concentrations up to 100 µg/mL. This is consistent with their intrinsic resistance due to outer membrane impermeability [36]. The selective antibacterial activity of *C. leptophloeos* stem bark extract can be attributed to its high phenolic content, particularly proanthocyanidins [23]. These compounds are capable of disrupting the peptidoglycan layer, damaging the cytoplasmic membrane, and interfering with protein and nucleic acid synthesis, which explains their effectiveness against Gram‐positive bacteria. In Gram‐negative organisms, however, the presence of an additional outer membrane composed of lipopolysaccharides acts as a permeability barrier, hindering the entry of many phenolic compounds, especially those of higher molecular weight. This structural difference justifies why extracts rich in phenolics typically display stronger activity against Gram‐positive pathogens and limited or absent activity against Gram‐negative strains [37]. Previous studies with *C. leptophloeos* extracts corroborate these findings, reporting antibacterial effects against Gram‐negative strains only at concentrations higher than 1000 µg/mL [19, 33]. According to [38], plant extracts are considered to have clinically important antibacterial activity when MIC values ​​are below 100 µg/mL. Although higher concentrations are often explored, the present study focused on concentrations ≤ 100 µg/mL, consistent with therapeutic relevance and potential for clinical application [39, 40].

The selective activity of HECL against Gram‐positive bacteria can be attributed to its high content of phenolic compounds, including procyanidins. Although these compounds have limited action against Gram‐negative organisms, they are known to exert multiple antibacterial mechanisms against Gram‐positive pathogens, such as disruption of cell wall and membrane integrity, inhibition of protein synthesis, and interactions with DNA that lead to bacterial death [41].

Our findings showed that parameters such as body weight, leukocyte counts, and acetylcholinesterase (AChE) activity provided insight into the systemic impacts of *C. leptophloeos*. while HECL partially maintained leukocyte counts during 5‐FU treatment, suggesting a potential immunomodulatory role, the interpretation of this effect is limited. Our analysis relied mainly on general hematological parameters without immune cell subset characterization (e.g., lymphocyte, neutrophil, monocyte profiles) or a broader cytokine panel beyond the selected mediators. In addition, HECL did not significantly prevent chemotherapy‐associated weight loss, which raises questions about its systemic protective capacity. These limitations highlight the need for more comprehensive immunological and physiological evaluations, such as flow cytometry, metabolic assays, and long‐term toxicity studies, to determine the therapeutic relevance of *C. leptophloeos* in reducing chemotherapy‐induced adverse effects,.hile the extract did not significantly prevent chemotherapy‐associated weight loss, the relative maintenance of leukocyte counts suggests a immunomodulatory effect [42].

Notably, treatment with *C. leptophloeos* at 10 mg/kg (HECL) preserved AChE activity, indicating that intestinal motility remains relatively maintained, which may help mitigate the severity of diarrhea or constipation. In contrast, intestinal mucositis induced by the chemotherapeutic agent 5‐fluorouracil (5‐FU) is typically associated with a significant increase in AChE activity, reflecting alterations in enteric nervous system function. This increased leads to an accumulation of AChE, which may result in dysregulation of the inflammatory response and exacerbation of tissue damage. Therefore, maintaining adequate AChE activity is crucial for balancing the inflammatory response during intestinal mucositis, contributing to the integrity and functionality of the gastrointestinal mucosa [43].

Treatment with *C. leptophloeos* at 10 mg/kg (HECL) significantly reduced IL‐6 levels, which may contribute to the preservation of AChE activity and, consequently, maintenance of intestinal motility. Elevated IL‐6 is known to disrupt enteric nervous system function, potentially leading to altered AChE activity and impaired gut motility. By reducing IL‐6, HECL helps normalize AChE activity, mitigating the risk of diarrhea or constipation in conditions of intestinal mucositis induced by 5‐fluorouracil (5‐FU). These findings are supported by previous studies demonstrating a correlation between proinflammatory cytokines and AChE‐mediated regulation of intestinal motility [44].

Biochemical markers (ALT, creatinine, and urea) remained within physiological limits across all treated groups, suggesting that HECL did not induce systemic toxicity at the tested doses. These findings align with previous toxicity studies, which reported no behavioral or biochemical alterations following high‐dose administration of *C. leptophloeos* (2000 mg/kg, p.o.) in rodents. The biochemical parameters (triglycerides, proteins, cholesterol, glucose, urea, creatinine, and aspartate aminotransferase) were not statistic significantly between the test and control groups (saline) [34].

Intestinal mucositis stands out as one of the most challenging complications arising from chemotherapy, particularly with the administration of 5‐FU, largely due to its multifaceted mechanisms encompassing oxidative stress, inflammation, and epithelial injury [5, 6]. In this study, the HECL demonstrated a protective effect on intestinal mucosa, especially at a dose of 10 mg/kg, as evidenced by the preservation of villi and crypts in duodenal sections. These findings are in line with previous observations that *Commiphora* species possess anti‐inflammatory properties [45], possibly tied to phenolic compounds and flavonoids [7]. Furthermore, the expression of MUC‐2 in HECL‐treated animals is indicative of enhanced mucus barrier function, which is critical for mitigating gut permeability and thus reducing the severity of mucositis [8, 46]. Although HECL treatment preserved intestinal architecture and increased MUC‐2 expression, thereby supporting improved barrier function, the exact molecular mechanisms underlying these effects remain incompletely defined. The phytochemical analysis confirmed a predominance of proanthocyanidins and flavonoids, both of which are known for antioxidant and anti‐inflammatory activities [24]; however, their individual contribution to the protective effects was not specifically dissected in vivo. Furthermore, although MUC‐2 was significantly upregulated, other markers of epithelial integrity (e.g., claudins, ZO‐1), oxidative stress (e.g., Nrf2/HO‐1), and inflammatory signaling pathways (e.g., NF‐κB, COX‐2, and iNOS) were not investigated. This limits definitive conclusions about the molecular basis of HECL's activity. Therefore, our findings should be considered as an initial step, and future studies are needed to integrate phytochemical bioavailability with mechanistic analyses of epithelial barrier preservation and immune modulation.

Research on other phenolic compounds suggests that they may modulate MUC‐2 expression, contributing to intestinal mucosal protection. For example, a study using a human colorectal adenocarcinoma cell line indicated that polyphenols can regulate the expression of MUC‐2, protecting against mucosal damage [47]. Additionally, vitexin, a flavonoid, was shown to dose‐dependently increase the expression of MUC‐2 in colonic tissues of mice with induced colitis [48].

 In vivo in the model of intestinal inflammation induced by 2,4‐dinitrobenzenesulfonic acid (DNBS) in mice when they were treated with *C. leptophloeos* was able to downregulate NF‐κB p65/COX‐2, mTOR, iNOS, and IL‐17, decrease levels of malondialdehyde and myeloperoxidase and cytokines TNF‐α, IL‐1β, and IL‐6 [15]. Moreover, the observed trend toward decreased MDA levels in our study, although not statistically significant, supports the hypothesis that compounds in HECL have the potential to reduce lipid peroxidation, a key process in the pathogenesis of intestinal injury [13].

In summary, the stem bark extract of *C. leptophloeos* exhibited a multifaceted protective effect against 5‐FU‐induced intestinal mucositis, likely mediated by its antibacterial, anti‐inflammatory, and barrier‐preserving properties. These results support its traditional use and underscore the need for further mechanistic and translational studies.

## 3 Conclusions

In summary, the hydroethanolic extract of *C. leptophloeos* stem bark (HECL) demonstrated promising pharmacological potential in a murine model of 5‐FU‐induced intestinal mucositis. The extract exhibited selective antibacterial activity against clinically relevant Gram‐positive pathogens and preserved intestinal architecture and mucosal integrity. These effects may be attributed to the predominance of proanthocyanidins and the presence of glycosylated flavonoids, which are likely involved in the extract's antioxidant, anti‐inflammatory, and immunomodulatory properties. Nevertheless, our findings must be interpreted with caution. The immunological analysis was based on general hematological parameters, without evaluation of immune cell subsets or a full cytokine profile, and HECL did not significantly prevent body weight loss. In addition, while MUC‐2 expression was increased, other critical markers of epithelial integrity and inflammatory signaling were not assessed. These limitations emphasize the need for future studies exploring immune cell dynamics, molecular signaling pathways, and long‐term safety to confirm the therapeutic relevance of *C. leptophloeos* in reducing chemotherapy‐induced toxicity. Collectively, these results support the ethnopharmacological use of *C. leptophloeos* and suggest its potential as an adjunctive therapeutic strategy for managing intestinal mucositis and associated bacterial infections in oncology settings. This study represents the first comprehensive evaluation of the dual therapeutic potential of *C. leptophloeos* bark extract as an adjuvant therapy to mitigate chemotherapy‐induced intestinal mucositis, providing scientific validation for its traditional use and paving the way for future drug development.

## 4 Experimental Section

### 4.1 Plant Material and Preparation of Hydroethanolic Extract

Stem barks from *C. leptophloeos* (Mart.) J.B. Gillett (Burseraceae) were collected in Altinho city, Pernambuco State, Brazil, in July 2018, at the following coordinates 8° 29 ‘32 “S and 36° 03’ 03” W. The plant material was botanically identified by Dr. Geraldo Mariz and specimens of *C. leptophloeos* stem barks were deposited at the Geraldo Mariz Herbarium of the Federal University of Pernambuco (voucher number UFPE 46,191). The permission to collect it was issued from System of Authorization and Information on Biodiversity (SISBIO/Brazil 35017), and by the Board of Management of Genetic Heritage of the Ministry of the Environment (CGEN/MMA—SISGEN/Brazil A618873). The plant name was confirmed using The World Flora Online database (www.worldfloraonline.org; accessed January 24, 2025).

The hydroethanolic extract from *C. leptophloeos* stem bark (HECL) was prepared according to Dantas–Medeiros et al. (2021) [34]. In summary, a portion of dried stem bark, (100 g) was resuspended in an ethanol:water (7:3, v/v) mixture in a proportion of 1:10 (w/v). The solvent was then evaporated at ≤40°C under reduced pressure with the aid of a rotary evaporator (Buchi‐Model V‐700, Altendorfer Str. 3, Essen, Germany). After the solvent (ethanol) was completely dried, the wet phase was lyophilized and stored at −20°C, resulting in a yield of 16.5% (16.5 g).

### 4.2 Analysis of Phytochemical Composition by Mass Spectrometry

The qualitative characterization by UPLC‐ESI‐IT‐MS was performed using an ultraperformance liquid chromatography system (Thermo Scientific) equipped with an Accela AS autosampler and an Accela 600 quaternary pump, coupled to an LTQ XL ion trap mass spectrometer with an electrospray ionization (ESI) source operating in negative ion mode (Thermo, San Jose, CA, USA). Chromatographic separation occurred on a Waters C18 reversed‐phase column (150 mm × 4.6 mm, 5 µm) maintained at ambient temperature. The column temperature was not actively controlled because the objective of the analysis was qualitative phytochemical profiling based primarily of MS/MS fragmentation patterns, for which minor temperature fluctuations do not significantly affect compound annotation or chromatographic performance.

The mobile phases consisted of water containing 0.1% formic acid (phase A) and methanol (phase B), with a linear gradient from 5% to 100% B over 10 min. Samples were injected in a volume of 10 µL, and the system operated at a flow rate of 300 µL/min. The mass spectrometer was configured with a capillary voltage of −40 V, spray voltage of 5.00 kV, source current of 5.09 µA, and a capillary temperature of 350°C.

For direct infusion, samples were introduced through a fused‐silica capillary maintained at 280°C, using the same LTQ XL system. Operating parameters included a spray voltage of 5.00 kV, capillary voltage of −47 V, and tube lens voltage of −226 V. Full scan mass spectra were acquired within an *m*/*z* range of 150–1500. MS/MS fragmentation analyses were performed on selected precursor ions using a collision energy of 30% and activation time of 30 ms. Data were processed using Xcalibur software (version 2.0).

It is important to note that chromatographic separation was used for metabolic profiling and precursor ion selection, while direct infusion was applied solely to enhance fragmentation signal intensity for qualitative structural annotation. Thus, compound annotation was based on interpretation of MS/MS fragmentation behavior, comparison with literature data for metabolites reported in the Commiphora genus, and previously isolated compounds from *C. leptophloeos* [17, 18].

### 4.3 Antibacterial Activity Assay

The antibacterial potential of HECL was determined through the agar dilution technique, following the protocols established by the National Committee for Clinical Laboratory Standards (NCCLS, 2000). HECL was initially solubilized in dimethyl sulfoxide (DMSO) and incorporated into Mueller–Hinton agar to obtain final concentrations ranging from 1.0 to 100 µg/mL. For preliminary screening, a concentration of 100 µg/mL was used. Each agar plate (20 mL) was prepared by combining 250 µL of the DMSO‐dissolved extract with 1.75 mL of sterile distilled water, followed by the addition of molten Mueller–Hinton medium. After solidification, the agar surfaces were inoculated using a Steers replicator with approximately 10^3^ CFU per spot of the test organisms.

The antimicrobial assay included a total of 90 bacterial strains, encompassing both reference strains and clinical isolates. Standard strains were obtained from recognized microbial repositories such as American Type Culture Collection/ATCC (USA), Collection of the Institut Pasteur/CIP, and Centre de Ressources Biologiques de l'Institut Pasteur/, CRBIP (France), while clinical isolates were categorized as E‐ and N‐, representing pathogens linked to respiratory, gastrointestinal, and urinary tract infections. The panel covered a wide range of Gram‐positive bacteria, including *Bacillus*, *Enterococcus*, *Listeria*, *Staphylococcus*, and *Streptococcus*, as well as Gram‐negative genera such as *Salmonella*, *Klebsiella*, *Enterobacter*, *Serratia*, *Pseudomonas*, *Yersinia*, *Vibrio*, *Citrobacter*, *Achromobacter*, and *Aeromonas*.

Ofloxacin served as the positive control, while DMSO at a final concentration of 1.25% was employed as the negative control. All plates were incubated aerobically at 37°C for 24 h. Ofloxacin was selected as the positive control due to its well‐established broad‐spectrum antibacterial activity against both Gram‐positive and Gram‐negative bacteria, allowing for reliable comparison of the antimicrobial potential of HECL with a standard reference drug.

The antibacterial assay against anaerobic *Streptococcus* strains was performed according to [16], using Wilkins–Chalgren agar. The incubation of plates was carried out under anaerobic conditions using Genbox Microaer sachets for 24 h at 37°C. All other steps, including sample preparation and microbial inoculation, followed the same experimental protocol. The minimum inhibitory concentration (MIC) was determined as the lowest concentration of HECL capable of completely preventing visible bacterial growth after incubation, based on visual comparison with appropriate positive and negative controls. Results are expressed as mean values accompanied by the standard deviation. Each test was conducted in triplicate (*n* = 3).

### 4.4 Intestinal Mucositis Experimental Model

Intestinal mucositis was induced as previously described [49]. Briefly, a single dose of 450 mg/kg of 5‐Fluorouracil (Libbs Pharmaceuticals LTDA, São Paulo, Brazil) was intraperitoneally administered and animals were euthanized 12th days later by overdose of ketamin (240 mg/kg) and xylazin (30 mg/kg).

### 4.5 Animals and Sample Size

Female Swiss mice (*Mus musculus*), aged approximately 8 weeks and weighing between 25 and 30 g, were maintained in polypropylene cages under standardized environmental conditions (temperature: 24°C ± 2°C; humidity: 50% ± 5%) with a 12‐h light/dark cycle. Animals had free access to standard chow and water throughout the study. The mice were sourced from the animal facility of the Biosciences Center at the Federal University of Rio Grande do Norte (UFRN), Brazil. All procedures were carried out in compliance with institutional and international ethical standards, following approval by the Ethics Committee on Animal Use of UFRN (Protocol No. 052/2023) and adhering to the ARRIVE guidelines and relevant regulatory frameworks.

The number of animals per group was determined according to the sample size formula *n* = DF/k + 1, which can be used for three common ANOVA designs applicable to animal studies, where k = number of groups, n = number of subjects per group, and DF = degrees of freedom [50]. In view of the ethical considerations that recommend sample size refinement in studies with animals, we chose to use the minimum number of animals (5 animals/group) to carry out experiments.

### 4.6 Experimental and Control Groups

To investigate the impact of HECL on 5 Fluorouracil ‐induced intestinal mucositis, 3 groups of 5 animals received once‐daily oral administration of HECL (doses: 0.5 or 1.0 or 10 mg/kg), starting 30 5‐Fluorouracil ‐FU injection. The control animals were divided into two control subgroups (*n* = 5/group): a group of healthy animals, not submitted to 5‐Fluorouracil ‐induced intestinal mucositis, that received once‐daily administration of saline solution until euthanasia and a single intraperitoneal injection of saline solution on day 1 (saline group/Negative Control/NC group) and a group of animals submitted to 5‐Fluorouracil‐induced intestinal mucositis that received once‐daily administration of saline solution from day 0 to day 3 (5FU group). All animals were euthanized 4 days after the 5 Fluorouracil injection.

The animals were monitored daily up to fourth day for signs of moribundity and mortality, such as lack of responsiveness to manual stimulation; immobility; and/or an inability to eat or drink.

### 4.7 Body Weight, Peripheral Blood Leucocyte Counts, and Hematological Analysis

Body weight and peripheral blood leucocyte counts were assessed to identify potential systemic toxicity associated with 5FU. Body weight was measured on the first and third days of the experiment. For comparison purposes, the weight of each group on the first day was set at 100%. On the third day, the body weight variation was expressed as an increase or decrease relative to the initial value. Before euthanasia, blood samples (20 µL) were collected from the heart puncture of anesthetized animals. These samples were then diluted in 380 µL of Turk's solution. Manual counting of total leukocytes was performed using a Neubauer chamber, with the results presented as the number of white blood cells per mm^3^ of blood. Alanine aminotransferase ALT, urea and creatinine.

### 4.8 Malonaldehyde Dosage (MDA)

The content of MDA, a product of lipid peroxidation, was investigated in the jejunal tissue samples (*n* = 5/group) as a marker of oxidative stress, as previously described [51]. The samples were diluted in Trizma buffer at a ratio of 1:5 (w/v). The mixtures were incubated in a water bath at 45°C for 40 min, followed by centrifugation at 2500 g for 5 min at 4°C. Subsequently, 300 µL of the supernatant was collected, and absorbance was measured at 586 nm. Quantification was performed by interpolation using a standard curve. The supernatants were distributed into microplates for the determination of malondialdehyde (MDA) levels. Absorbance readings were taken at 586 nm, and results were expressed as nanomoles of MDA per milliliter of plasma or per milligram of tissue.

### 4.9 Acetylcholinesterase Activity and Fecal Retention in the Intestine

Fecal retention in each group was assessed at the time of euthanasia. The amount and extent of retained feces were assessed. We established a score from 0 to 2, where 0 means no retention; 1 means low retention; and 2 means moderate to severe retention.

The determination of acetylcholinesterase (AChE) activity in colon samples was carried out following the protocol described by Ellman et al. [52]. Colon segments (*n* = 5/group) were homogenized in sodium phosphate buffer (pH 7.0) at a ratio of 10 mg tissue per 100 µL of buffer and then centrifuged at 5000 rpm for 20 min at 4°C. For the enzymatic assay, 500 µL of phosphate buffer, 895 µL of distilled water, 50 µL of 5,5’‐dithio‐bis‐(2‐nitrobenzoic acid/DTNB solution (prepared by dissolving 0.04 g DTNB and 0.015 g sodium bicarbonate in 10 mL phosphate buffer), and 5 µL of tissue homogenate were transferred into a glass cuvette. After calibrating the spectrophotometer with the blank solution, 50 µL of acetylthiocholine iodide solution (0.073 g dissolved in 5 mL distilled water) was added to initiate the reaction. Absorbance readings were recorded at 412 nm at 0, 30, 120, and 360 s to monitor enzymatic activity.

### 4.10 Cytokine Assay (IL‐1β and IL‐6)

The IL‐1β and IL‐6 levels were determined from jejunum samples (*n* = 5/group), which were stored at −80°C until required for this assay. The samples were homogenized and processed as previously described [53]. The concentrations of IL‐1β, IL‐6, and TNF‐α in the samples were determined using a commercial ELISA kit (R&D Systems, Minneapolis, MN, USA). Briefly, microtiter plates were coated overnight at 4°C with antibodies against IL‐1β (detection range: 62.5–4000 pg/mL; sensibility or lower limit of detection: 12.5 ng/mL of recombinant mouse IL‐1β), IL‐6 (detection range: 125–8000 pg/mL). After blocking the plates, the samples and standard at various dilutions were added in duplicate and incubate at 4°C for 24 h. After washing the plates (three times with buffer) biotinylated polyclonal anti‐IL‐1β or anti‐TNF‐α, diluted 1:1000 with assay buffer 1% BSA, was added to the wells. After further incubation at room temperature for 1 h, the plates were washed and streptavidin‐HRP, diluted 1:5000, was added to each well. The chromogenic reagent *O*‐phenylenediamine was added 15 min later and the plates were incubated in the dark for 15 min. The enzymatic reaction was interrupted with H_2_SO_4_ and the absorbance was measured at 490 nm using UV–vis spectrophotometry. The results are expressed as pg/mL [54].

### 4.11 Histopathological Analysis

After euthanasia, full‐thickness (*n* = 5/group) samples from different regions of the small intestine (duodenum, jejunum, and ileum) and colon, including mucosa, submucosa, muscularis, and serosa layers, were collected. The tissues were fixed in 10% neutral buffered formalin, followed by dehydration and paraffin embedding for histological and immunohistochemical analyses. Serial sections (5 µm thick) were stained with hematoxylin and eosin (H&E) and examined under a light microscope at 200× magnification. The extent of mucosal injury was assessed in a blinded manner using a modified version of the histopathological grading system originally described by Macpherson and Pfeiffer [55].

### 4.12 Immunohistochemical Analysis

Intestinal tissue sections (duodenum, *n* = 5/group) with a thickness of 4 µm were obtained from paraffin‐embedded samples. Following deparaffinization, antigen retrieval was performed by incubating the slides in citrate buffer (pH 6.0) at 95°C for 20 min. Endogenous peroxidase activity was blocked using 3% hydrogen peroxide for 10 min. The sections were then incubated for 2 h with primary antibodies against MUC‐2 or occludin (Santa Cruz Biotechnology). Detection was performed using a polymer‐based detection system (K4061, Dako) for 30 min, followed by visualization with a diaminobenzidine (DAB) and hydrogen peroxide solution (Dako). Negative controls were prepared by omitting the primary antibody and incubating the sections with antibody diluent only. Immunoreactivity was quantified from digital images captured at 400× magnification in at least ten randomly selected fields per section (four specimens per group). The percentage of immunopositive area was calculated by dividing the DAB‐positive stained area (in pixels) by the total number of pixels in each tissue image, and multiplying by 100, as previously described [56].

### 4.13 Statistical Analysis

Data are presented as mean ± standard error of the mean (SEM) and illustrated as bar graphs. Histological scores are expressed as median with interquartile range (IQR). Error bars represent the 25th–75th percentiles. Owing to the small sample size (*n* = 5 animals per group) and the absence of a guarantee of normal data distribution, nonparametric statistical tests were applied. Statistical analysis was performed using Kruskal–Wallis test followed by Dunn's post hoc test. *p* < 0.05, a significance level of 5% (*p* < 0.05) was adopted for all statistical analyses. All analyses were conducted using GraphPad Prism software (version 6.01).

## Funding

This research was supported by the Brazilian research funding agencies: Conselho Nacional de Desenvolvimento Científico e Tecnológico (CNPq) and Coordenação de Aperfeiçoamento de Pessoal de Nível Superior (CAPES). Renato Dantas‐Medeiros was a Junior Postdoctoral Fellow at CNPq at the Federal University of Rio Grande do Norte (UFRN) [grant number 151486/2024‐7]. Silvana Maria Zucolotto is a CNPq research productivity fellow [grant number 313727/2020‐1]. Additional funding was provided by CNPq [grant numbers 303915/2023‐4, 401672/2023‐9], and INCT iCEIS [CNPq 406264/2022‐8]; INCT‐BIOFOTO BUCAL CNPq: 408830/2024‐7.

## Conflicts of Interest

The authors declare no conflict of interest.

## Data Availability

Data available on request from the authors.
